# Parallel circuits control temperature preference in *Drosophila* during ageing

**DOI:** 10.1038/ncomms8775

**Published:** 2015-07-16

**Authors:** Hsiang-Wen Shih, Chia-Lin Wu, Sue-Wei Chang, Tsung-Ho Liu, Jason Sih-Yu Lai, Tsai-Feng Fu, Chien-Chung Fu, Ann-Shyn Chiang

**Affiliations:** 1 Institute of Biotechnology, National Tsing Hua University, Hsinchu 30013, Taiwan.; 2 Department of Biochemistry and Graduate Institute of Biomedical Sciences, College of Medicine, Chang Gung University, Taoyuan 33302, Taiwan.; 3 Department of Medical Research, Chang Gung Memorial Hospital, Taoyuan 33305, Taiwan.; 4 Department of Power Mechanical Engineering, National Tsing Hua University, Hsinchu 30013, Taiwan.; 5 Department of Applied Chemistry, National Chi Nan University, Nantou 54561, Taiwan.; 6 Brain Research Center, National Tsing Hua University, Hsinchu 30013, Taiwan.; 7 Genomics Research Center, Academia Sinica, Nankang, Taipei 11529, Taiwan.; 8 Department of Biomedical Science and Environmental Biology, Kaohsiung Medical University, Kaohsiung 80780, Taiwan.; 9 Kavli Institute for Brain and Mind, University of California at San Diego, La Jolla, California 92093-0526, USA.

## Abstract

The detection of environmental temperature and regulation of body temperature are integral determinants of behaviour for all animals. These functions become less efficient in aged animals, particularly during exposure to cold environments, yet the cellular and molecular mechanisms are not well understood. Here, we identify an age-related change in the temperature preference of adult fruit flies that results from a shift in the relative contributions of two parallel mushroom body (MB) circuits—the β′- and β-systems. The β′-circuit primarily controls cold avoidance through dopamine signalling in young flies, whereas the β-circuit increasingly contributes to cold avoidance as adult flies age. Elevating dopamine levels in β′-afferent neurons of aged flies restores cold sensitivity, suggesting that the alteration of cold avoidance behaviour with ageing is functionally reversible. These results provide a framework for investigating how molecules and individual neural circuits modulate homeostatic alterations during the course of senescence.

## Abstract (web summary)

The capacity for thermoregulation deteriorates with age, particularly in cold environments. Here the authors demonstrate in *Drosophila* that age-related changes in cold avoidance result from a shift in the relative contribution of two parallel mushroom body circuits that are modulated by dopamine.

## Graphical abstract

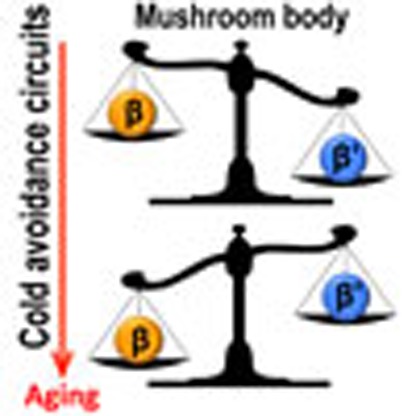

The capacity for thermoregulation is ubiquitous and is found in animals ranging from flies to humans^1^. Although the different ways in which animals regulate their body temperature (for example, homoeothermic mechanisms in the central nervous system of endotherms such as mammals and birds versus behavioural thermoregulation in reptiles) allow different species to tolerate environments of various temperature ranges, all animals must avoid extreme hot or cold temperatures that are harmful and damaging to life. Therefore, the ability to maintain body temperatures within an optimal range is essential for the survival of all animals.

The capacity for behavioural thermoregulation changes as animals age, particularly during exposure to cold environments^2^,^3^. As ageing significantly alters the structure and function of the nervous system^4^, such age-related changes in thermal sensitivity and regulation might be expected^5^,^6^. In mammals, age-dependent changes in thermoregulation are linked with age-related degradation in the dopamine system. For example, in rats, the activity of tyrosine hydroxylase (TH, an enzyme critical for dopamine biosynthesis) declines in the brain with age, leading to a decreased ability to cope with cold stress^7^,^8^. However, the underlying molecular and circuit-level mechanisms are not well understood.

We have been investigating thermoregulation in the adult fruit fly, *Drosophila melanogaster*, because it affords a rare opportunity to clarify the mechanisms that underlie a crucial neurobehavioural function at the molecular, cellular and circuit levels. Accumulating evidence suggests that, as in mammals, age-related changes in the dopamine system also underlie changes in behavioural thermoregulation in *Drosophila*. First, temperature preference in the fruit fly is highly variable following eclosion but becomes stable after 5 days^9^, suggesting that it changes as adults age. Second, whole-body dopamine levels as well as TH immunoreactivities in specific neuron clusters in the brain decline with advancing age^10^. Third, a brain region called the mushroom body (MB) is required to regulate temperature preference^11^, and dopamine receptors (DopRs) in the Kenyon cells (KCs)—the intrinsic neurons of the MB—are required for behavioural thermoregulation mediated by cold avoidance^12^. Collectively, these data suggest that alteration of dopamine signalling in the MB plays an important role in age-related changes of temperature preference in *Drosophila*.

Here we first characterize changes in temperature preference with advancing age. We then detail the underlying cellular and molecular mechanisms that lead to such changes in adult fruit flies by identifying dopamine signalling within two separate MB circuits. Finally, we show that a shift in the relative contributions of these two parallel MB circuits results in reduced cold avoidance in aged individuals. Our results indicate that as flies age, the β-circuit increasingly contributes to cold avoidance as reductions in dopamine signalling and functional responses of neurons to cold stimuli in the β′-circuit increasingly lead to an age-dependent decline in cold avoidance.

## Results

### Reduced cold avoidance in aged fruit flies

**Figure 1:**
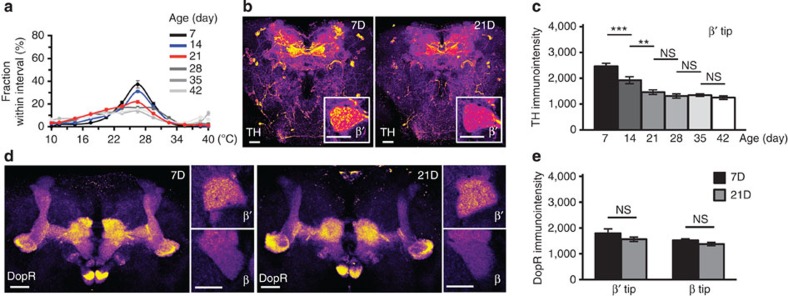
Age-dependent changes in temperature preference and brain tyrosine hydroxylase (TH) levels. (**a**) Temperature preference of wild-type flies at different ages (7–42 days). (**b**) TH immunostaining in the brain of 7- (7D) and 21-day-old (21D) flies. Inset, magnified view of MB β′-tip. (**c**) Fluorescent intensity of TH**-**immunopositive signals in MB β′-tip of flies at different ages. (**d**) An optical section through β′-/β-lobes indicating dopamine receptor (DopR) immunostaining in 7D (left) and 21D (right) flies. Insets, magnified views of MB β′-tip (top) and β-tip (bottom). (**e**) Fluorescent intensity of DopR-immunopositive signals in MB β′-tip and β-tip of 7D and 21D flies. Scale bar, 20 μm. Values represent mean±s.e.m. (*n*=12). ***P<0.001; **P<0.01; NS, not significant.

To clarify the time course of age-dependent changes in temperature preference throughout the lifespan of adult *Drosophila*, we allowed wild-type flies of different ages (7–42 days) to move freely for 30 min on a plate with a thermal gradient in which the temperature ranged from 10 to 40 °C (Supplementary Fig. 1). We found that young flies (7–14 days) exhibit a stronger preference than old flies (21–42 days) to stay within an intermediate thermal region between 22 and 28 °C. At cooler regions below 22 °C, the fraction of old flies was significantly higher than that of young flies (Fig. 1a). This is not due to altered mobility, because aged flies display normal locomotor activity^13^.

Given that normal temperature preference requires DopRs in the MB^12^, we asked whether TH and DopR levels change in the aged MB. In young flies, TH-immunopositive signals at the tip of the β′-lobe were strongest among all MB regions (Fig. 1b; Supplementary Fig. 2). Among all MB lobes, TH levels decreased most significantly at the β′- and β-tips between 7 and 14 days of age. However, further reduction occurred only at the β′-tip between 14 and 21 days of age (Fig. 1c), a period during which cold avoidance began to decline. In contrast, DopR-immunopositive signals at the β′- and β-tips remained at similar levels at least until day 21 (Fig. 1d,e). Subsequently, we used 7- and 21-day-old flies as representatives of young and aged flies, respectively, to address whether dopamine signalling within the MB β′-tip contributes to the age-dependent decline in cold avoidance.

### Branch-specific functional calcium response in MB KCs

**Figure 2:**
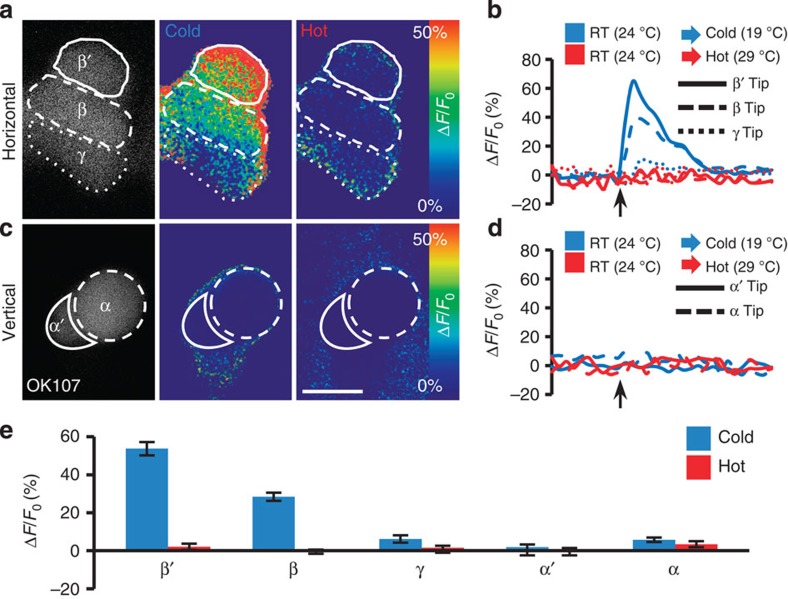
Functional responses of MB KCs to temperature stimuli. (**a**) Representative GCaMP1.6 response at tips of three horizontal lobes. (**b**) Temporal changes in GCaMP1.6 fluorescence to temperature stimuli in **a**. (**c**) Representative GCaMP1.6 response at tips of two vertical lobes. (**d**) Temporal changes in GCaMP1.6 fluorescence to temperature stimuli in **c.** Left, basal GCaMP1.6 expression; middle, response to cold stimulus (19 °C); right, response to hot stimulus (29 °C). (**e**) Quantification. The arrows indicate the time points at which the temperature stimulus was applied. Scale bar, 20 μm. Values represent mean±s.e.m. (*n*≥15).

The MB is composed of three major subsets of KCs whose axons bifurcate to constitute the αβ-, α′β′- and γ-lobes. More specifically, the β-, β′- and γ-lobes make up the horizontal branch of the MB, and the α- and α′-lobes form the vertical branch. We first asked how KCs respond to temperature changes by using the calcium reporter GCaMP^14^. Calcium imaging in *OK107-GAL4*>*UAS-GCaMP1.6* flies indicated that the MB exhibits a branch-specific calcium response to cold. In the horizontal branch, the β′- and β-lobes showed a robust increase in GCaMP fluorescence in response to cold stimuli (24 °C→19 °C), whereas the γ-lobe showed minimal response (Fig. 2a). The maximum fluorescence increased beyond baseline (Δ*F*/*F*_0_) by up to ∼60% for the β′-lobe and ∼40% for the β-lobe (Fig. 2b), but remained constant for the α- and α′-lobes of the vertical branches (Fig. 2c,d). The mean GCaMP fluorescence in response to cold stimuli consistently increased by ∼50% for the β′-lobe and ∼25% for the β-lobe, but remained constant in other lobes (Fig. 2e). Hot stimuli (24 °C→29 °C) did not elicit a response in any of the MB lobes (Fig. 2a–e). These data indicate that KCs exhibit branch-specific neural activity (that is, subregional activity within a single neuron). This resembles the branch-specific calcium response that occurs exclusively in the α-lobe of the vertical branch, and not in the β-lobe of the horizontal branch, after flies are associatively conditioned to avoid an odour paired with an electric shock^15^.

### The extrinsic neurons of β′- or β-lobe for cold avoidance

To identify the contributions of neurons in the β′- and β-lobes to cold avoidance, we conducted a pilot analysis of GAL4 lines containing MB β′- or β-extrinsic neurons. We first tested a set of 12 GAL4 lines containing MB β′-extrinsic neurons (Supplementary Fig. 3). Silencing neural activity with an inward rectifier potassium channel (Kir2.1)^16^ or tetanus toxin (TNT)^17^ in protocerebral anterior media (PAM)-M9 neurons (that is, *Ddc*-, *VT6202*- and *VT19841-GAL4s*) and MB-M4 neurons^16^ (that is, *VT41043*- and *VT44170-GAL4s*) impaired cold avoidance (Supplementary Fig. 3b). We also examined another collection of nine GAL4 lines containing MB β-extrinsic neurons. Disrupting neural activity in PAM-M8 neurons^19^,^20^ (that is, *0279-GAL4*) and MB-M10 neurons (that is, *275Y*-, *NP0243*-, *VT0765*- and *VT25781-GAL4s*) impaired cold avoidance (Supplementary Fig. 4). In accordance with existing MB extrinsic cell nomenclature^18^,^19^,^20^, we defined the two sets of dopaminergic PAM neurons innervating the b0-tip and b-lobe as PAM-M9 and PAM-M8, respectively. Dopaminergic PAM neurons, which comprise B110MB afferent neurons^21^ that extend their axon terminals to the horizontal branches of the MB, are implicated in processing reward memory in fruit flies^19^,^20^,^21^,^22^. In contrast, the function of MB-M4 and MB-M10 neurons (named by us following PAM-M9) remains largely unknown.

**Figure 3:**
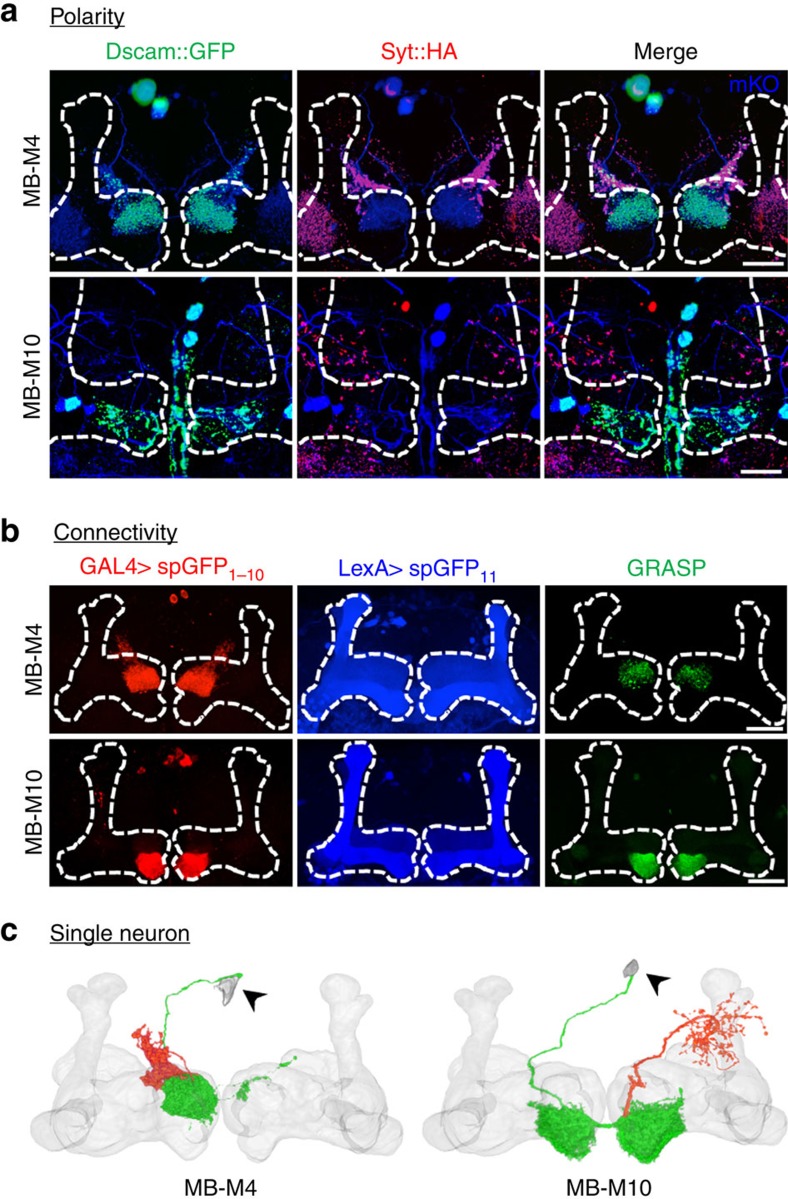
Directionality of connections of MB-M4 and MB-M10 neurons with MB. (**a**) Postsynaptic regions (green) and presynaptic terminals (red) of MB-M4 and MB-M10 neurons under mKO (monomeric Kusabira Orange) expression (blue) driven by *VT41043-GAL4* and *VT0765-GAL4*, respectively. (**b**) Signals of green fluorescent protein reconstitution across synaptic partners (GRASP) in the MB horizontal lobes (green) contributed by connections between KCs (blue) and MB-M4 neurons (red, upper panel) or between KCs (blue) and MB-M10 neurons (red, lower panel). (**c**) Morphology and innervation patterns of individual MB-M4 and MB-M10 neurons in relation to MB volume model. Individual MB-M4 and MB-M10 neurons were derived from FLP-out labelling of *VT41043-GAL4* and *VT0765-GAL4*, respectively. Dendrites (green) and axons (red) were assigned based on polarity analysis. Arrowhead, cell body. Scale bar, 20 μm.

To verify the information flow of MB-M4 and MB-M10 neurons, we performed polarity labelling with the presynaptic marker syt::HA^23^ and the postsynaptic marker Dscam::GFP^24^. The results showed that both MB-M4 and MB-M10 neurons gave rise to dendrites in the MB, indicating their roles as efferent neurons (Fig. 3a). Positive labelling of green fluorescent protein reconstitution across synaptic partners (GRASP)^25^ with spGFP_11_ expressed in all KCs and spGFP_1–10_ expressed in the MB efferent neurons suggested that MB-M4 and MB-M10 neurons are in close contact with the KCs (Fig. 3b). We therefore used FLP-out labelling^26^ to visualize the morphology and innervation pattern of individual MB-M4 and MB-M10 neurons (Fig. 3c). Each MB-M4 neuron had extensive dendritic arborizations at the contralateral β′-tip, but few extended arborizations at the ipsilateral β′-tip; in addition, its axon projected to the superior dorsofrontal protocerebrum (SDFP). Each MB-M10 neuron extended dendrites to both β-tips, and its axon projected to regions surrounding the ipsilateral vertical lobes.

### Functional calcium response to cold in the MB during ageing

**Figure 4:**
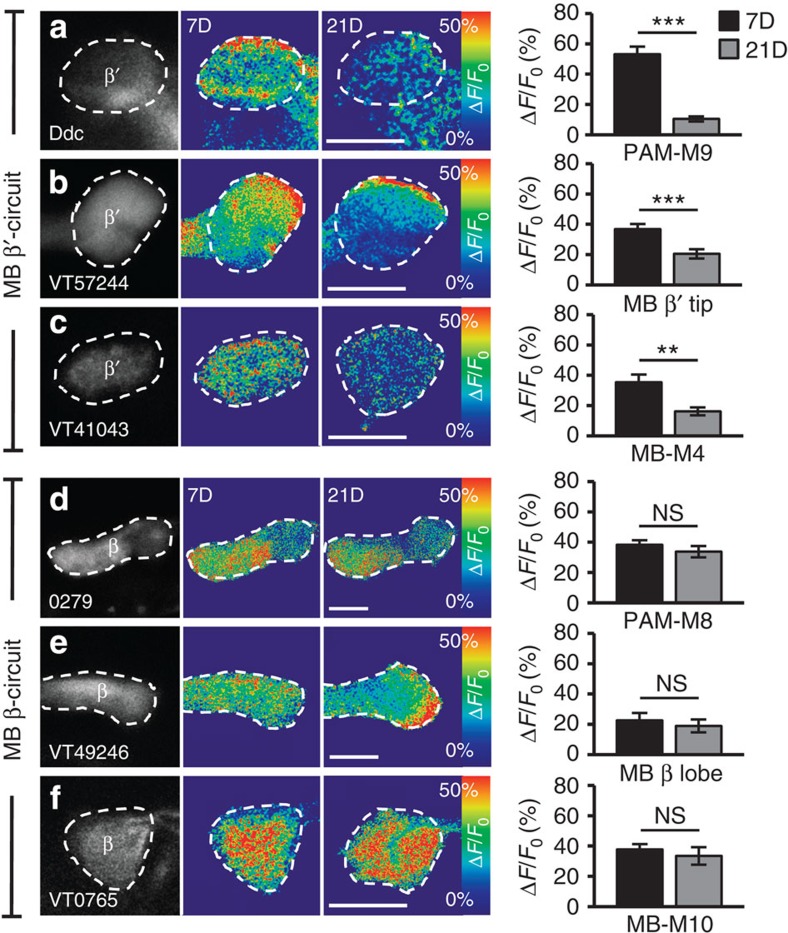
Functional responses of β′- and β-circuits to cold stimuli in young and aged flies. (**a**–**f**) GCaMP1.6 response to cold stimuli in PAM-M9 neurons (**a**), MB α′β′-KCs (**b**) and MB-M4 neurons (**c**) of MB β′-circuit. GCaMP1.6 response to cold stimuli in PAM-M8 neurons (**d**), MB αβ-KCs (**e**) and MB-M10 neurons (**f**) of MB β-circuit. Right, quantitative GCaMP fluorescence responses (Δ*F*/*F*_0_) to cold in young (7D) and aged flies (21D). Scale bar, 20 μm. Values represent mean±s.e.m. (*n*≥13). ***P<0.001; **P<0.01; NS, not significant.

Having identified the input and output neurons of the β′- or β-circuits, we next asked how their responses to cold stimuli change with ageing. Functional calcium imaging indicated that, as the flies aged, the GCaMP1.6 responses to cold stimuli decreased from ∼55 to ∼10% in PAM-M9 neurons (Fig. 4a), from ∼40 to ∼20% in the β′-branch of KCs (Fig. 4b) and from ∼40 to ∼20% in MB-M4 neurons (Fig. 4c). Surprisingly, the cold responses remained at a similar level in PAM-M8 neurons (Fig. 4d), the β-branch of KCs (Fig. 4e) and MB-M10 neurons (Fig. 4f) during the course of ageing. Collectively, these data suggest the existence of an age-dependent circuit and an age-independent circuit in the MB β′- and β-branches, respectively. The impairment of cold avoidance by surgical ablation or genetic manipulation suggests the existence of cold sensors in the third antennal segment^27^,^28^. Intriguingly, ablation of both antennae did not affect the responses to cold stimuli in PAM-M9, MB-M4, PAM-M8 or MB-M10 neurons (Supplementary Fig. 5a–d, respectively), indicating the existence of other cold sensors.

### Inhibiting neural activity in β′- or β-impairs cold avoidance

**Figure 5:**
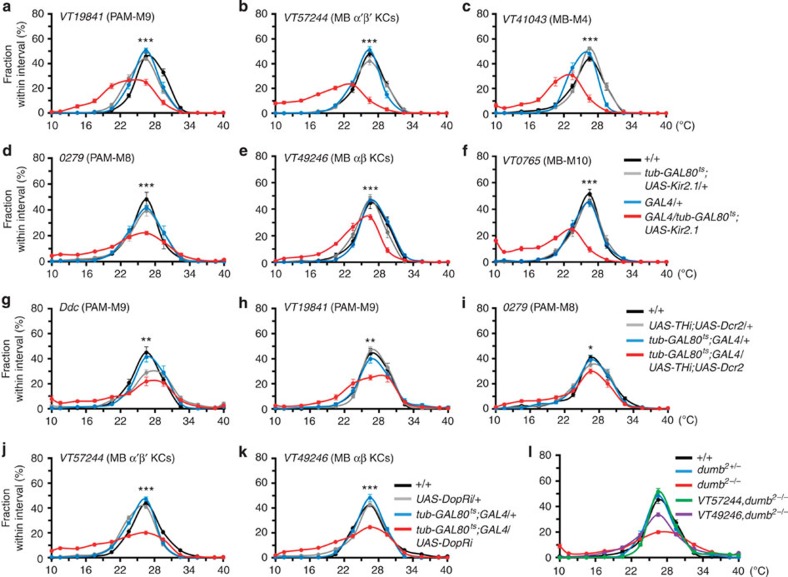
Neural activities in both β′- and β-circuits contribute to normal temperature preference in young flies. (**a**–**c**) Inhibiting neural activity of β′-circuit in PAM-M9 neurons under *VT19841-GAL4* (**a**), MB α′β′-KCs under *VT57244-GAL4* (**b**) and MB-M4 neurons under *VT41043-GAL4* (**c**). (**d**–**f**) Inhibiting neural activity of the β-circuit in PAM-M8 neurons under *0279-GAL4* (**d**), MB αβ-KCs under *VT49246-GAL4* (**e**) and MB-M10 neurons under *VT0765-GAL4* (**f**). (**g**–**i**) Downregulating TH levels in PAM-M9 neurons under *Ddc-GAL4* (**g**), *VT19841-GAL4* (**h**) and PAM-M8 neurons under *0279-GAL4* (**i**). (**j**,**k**) Downregulating DopR levels in α′β′-KCs under *VT57244-GAL4* (**j**) or in αβ-KCs under *VT49246-GAL4* (**k**). (**l**) Expressing DopR in α′β′-KCs or αβ-KCs in homozygote *dumb*^*2*^ mutant flies. Values represent mean±s.e.m. (*n*≥12). ***P<0.001; **P<0.01; *P<0.05.

To investigate the molecules responsible for the physiological and behavioural changes in cold avoidance, we then genetically manipulated the neural activities of the β′- and β-circuits. Adult stage-specific expression of Kir2.1 by temporal control of temperature-sensitive GAL80 inhibition^29^ on 11 independent GAL4 lines (Fig. 5a–f; Supplementary Fig. 6a–e) confirmed that silencing the components of either the β′- or β-circuit impaired cold avoidance in young flies. Acutely downregulating either dopamine levels with TH RNA interference (RNAi) in PAM-M9 (Fig. 5g,h) and PAM-M8 neurons (Fig. 5i), or DopR levels with DopR RNAi in α′β′-KCs (Fig. 5j; Supplementary Fig. 6f) and αβ-KCs (Fig. 5k; Supplementary Fig. 6g) consistently impaired cold avoidance. Restoring DopR levels by driving *UAS-DopR* expression in α′β′-KCs or αβ-KCs fully or partially rescued cold avoidance in *dumb*^*2*^ mutants (Fig. 5l). The efficacies of TH RNAi (Supplementary Fig. 7a,b), DopR RNAi (Supplementary Fig. 7c) and DopR overexpression in *dumb*^*2*^ mutants (Supplementary Fig. 7d) were confirmed. The behavioural results and functional calcium responses of MB KCs (Fig. 2a–e) suggest that the β′-circuit plays a dominant role in the cold avoidance of young flies.

### Two independent signals co-regulate cold avoidance

**Figure 6:**
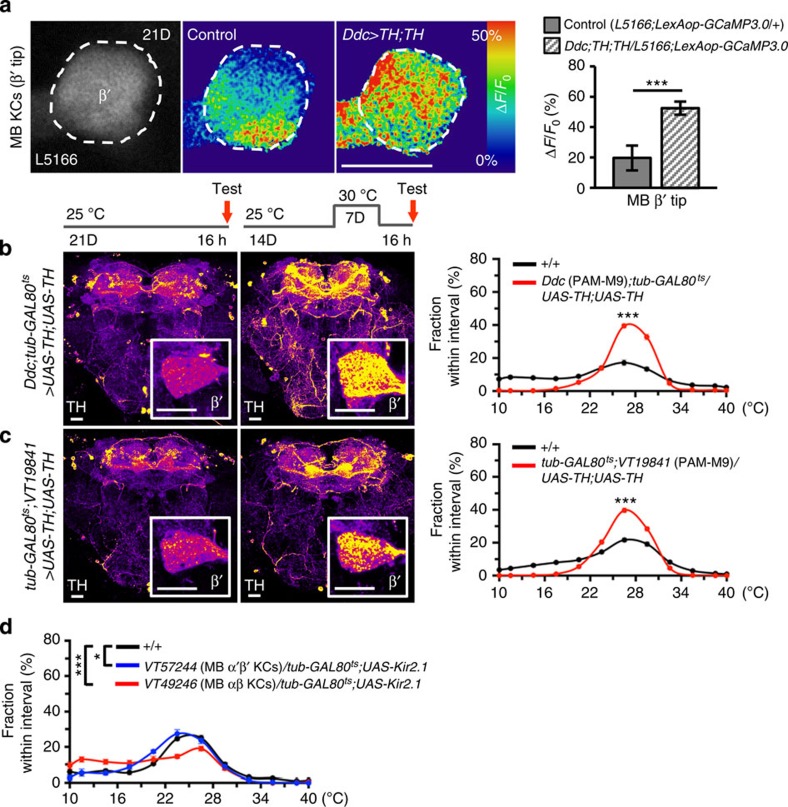
Dopamine signalling in the β′-circuit controls age-dependent cold avoidance. (**a**) GCaMP3.0 responses (Δ*F*/*F*_0_) of α′β′-KCs in β′-tip to cold increased in aged flies with TH overexpressed in PAM-M9 neurons (*n*=28) relative to aged control flies (*n*=17). Right, quantitative changes in GCaMP3.0 fluorescence. (**b**,**c**) Overexpressing TH in PAM-M9 neurons under *Ddc-GAL4* (**b**) and *VT19841-GAL4* (**c**) restored cold avoidance in aged flies (21D) relative to control flies of the same age (*n*≥20). Left, elevated TH-immunopositive signals after GAL80 removal. Inset, magnified view of MB β′-tip. Right, changes in temperature preference. (**d**) Effects of inhibiting neural activity in α′β′- and αβ-KCs in aged flies in comparison with wild-type flies. Scale bar, 20 μm. Values represent mean±s.e.m. (*n*≥12). ***P<0.001; *P<0.05.

To test whether the loss of cold sensitivity is functionally reversible, we manipulated the β′-circuit and observed the changes in the functional calcium response and behaviour of aged flies. When we overexpressed TH in the dopaminergic PAM-M9 neurons, the KCs downstream of their β′-terminals responded to cold with greater increases in GCaMP3.0 fluorescence (∼50%) than those in aged wild-type flies (∼20%) (Fig. 6a). In addition, these TH-overexpressed flies exhibited a pattern of temperature preference nearly identical to that of young individuals in comparison with that of aged wild types (Fig. 6b,c). These data suggest that the reduced cold avoidance in aged flies is functionally reversible.

Given that cold avoidance declines markedly with ageing (Fig. 1a) and young flies require normal neural activities in both MB β′- and β-circuits (Fig. 5a–f; Supplementary Fig. 6a–e), we wondered whether inhibiting neural activity in the β′- or β-circuit would further disrupt cold avoidance in aged flies. When we silenced neural activity in α′β′- or αβ-KCs with Kir2.1 in aged flies, only those with disrupted αβ-KCs showed fully impaired cold avoidance, which was far more severe than that of flies with disrupted α′β′-KCs or aged wild types (Fig. 6d). This suggests that the β-circuit increasingly assumes a greater role in maintaining cold avoidance as flies age.

### Mechanism underlying MB KC activity modulation by dopamine

**Figure 7:**
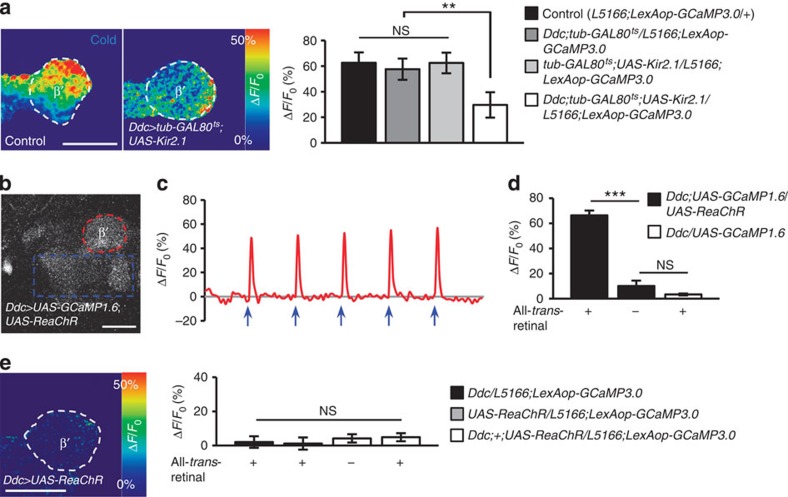
Effects of bi-directional manipulations of PAM-M9 activity. (**a**) GCaMP3.0 responses (Δ*F*/*F*_0_) of α′β′-KCs in β′-tip to cold stimuli in young control flies and in young flies in which PAM-M9 neurons were silenced using *UAS-Kir2.1.* Right, quantification of GCaMP fluorescence responses (Δ*F*/*F*_0_) in α′β′-KCs at the β′-tip. (**b**) Responses of PAM-M9 neurons to ReaChR activation were monitored by two-photon laser-scanning microscopy. In flies that expressed both GCaMP1.6 and ReaChR under *Ddc-GAL4*, the region including the cell bodies and fibres (dotted blue line) was activated by a 561-nm laser, and the fibres at β′-tip were recorded (dotted red line). (**c**) Temporal changes in GCaMP1.6 fluorescence to ReaChR activation of the β′-tip (region surrounded by the dotted red line in ‘**b**') when 5 pulses of the 561-nm laser (blue arrows) were delivered after every 20th scan to the region surrounded by the dotted blue line in ‘**b**'. (**d**) Quantification of GCaMP fluorescence responses (Δ*F*/*F*_0_) of β′-tip in ‘**b**' under each genetic manipulation. (**e**) GCaMP3.0 responses of α′β′-KCs in β′-tip in which *Ddc-GAL4* neurons were optogenetically activated using *UAS-ReaChR*. Scale bar, 20 μm. Values are presented as mean±s.e.m. (*n*≥10). ***P<0.001; **P<0.01; NS, not significant.

Finally, we asked whether the dopaminergic PAM neurons link the sensory input to the MB KCs or form part of a separate pathway that modulates the activity of MB KCs. To test this, we silenced PAM-M9 neurons in young flies using Kir2.1 and found that MB KCs at the β′-tip remained responsive to cold stimuli, although the magnitude of the GCaMP3.0 response was lower than that for MB KCs in control flies (Fig. 7a). Furthermore, we optogenetically activated PAM-M9 neurons with a red-shifted channelrhodopsin (ReaChR)^30^, and recorded the functional response of MB KCs at the β′-tip. To test the feasibility of activating a cluster of neurons and recording the responses of its putative downstream structures, we expressed both GCaMP1.6 and ReaChR under *Ddc-GAL4*. When we activated a portion of the *Ddc-GAL4* neurons, the GCaMP1.6 fluorescence intensity increased markedly in the remaining portions of the *Ddc-GAL4* neurons, suggesting the feasibility of this manipulation (Fig. 7b–d; Supplementary Movie). Activation of PAM-M9 neurons in this manner under *Ddc-GAL4*, however, failed to activate MB KCs at the β′-tip (Fig. 7e). Together these results support the idea that dopaminergic PAM-M9 neurons act as a parallel modulatory pathway modulating the calcium response of MB KCs to sensory inputs of cold stimuli (Supplementary Fig. 8).

## Discussion

In this study, we showed that in the fly brain, the α′β′- and αβ-KCs of the MB exhibit branch-specific activity in the β′- and β-lobes to cold, but not hot, stimuli (Fig. 2a–d). Polarity and GRASP assays indicated that the dendrites of MB-M4 and MB-M10 connect to the MB β′- and β-lobes, respectively, and that they receive signals directly from the α′β′- and αβ-KCs (Fig. 3). Functional calcium responses to cold stimuli declined markedly in PAM-M9 neurons, the β′-tip of KCs and MB-M4 neurons, but remained at similar levels in PAM-M8 neurons, the β-lobe of KCs and MB-M10 neurons in aged flies, suggesting that two independent MB circuits co-regulate cold sensitivity (Fig. 4a–f). Flies showed a substantial reduction in cold avoidance by genetically silencing activity along the PAM-M9→α′β′-KCs→MB-M4 or PAM-M8→αβ-KCs→MB-M10 pathways, suggesting that both the β′- and β-circuits of the MB are required for normal cold avoidance (Fig. 5a–f). RNA-mediated downregulation of TH level in PAM-M9 neurons caused a greater reduction in cold avoidance than did the same manipulation in PAM-M8 (Fig. 5g–i). Similarly, disrupting neural activity in α′β′-KCs led to a more severe reduction in cold avoidance than did the same manipulation in αβ-KCs (Fig. 5b,e), indicating that the β′-circuit may play a major role in temperature preference through a dopamine signalling pathway in young flies. However, the same manipulation led to the opposite result in aged flies (Fig. 6d). This suggests that as flies age, the β-circuit plays an increasingly greater role than the β′-circuit in cold avoidance. Therefore, our results reveal two parallel brain circuits—the β′- and β-circuits in the MB of adult fruit flies (Supplementary Fig. 8a,b)—that together control temperature preference in young adults, but whose relative contributions become increasingly biased towards the β-circuit with advancing age (Supplementary Fig. 8c). Furthermore, bi-directional manipulations of PAM-M9 activity suggest that the dopaminergic neurons act via a separate pathway to modulate the calcium responses of MB KCs to cold stimuli (Fig. 7).

The reduction of TH and dopamine levels in the brain is a common feature of ageing in both *Drosophila* and mammals^10^,^31^. In mammals, an age-dependent inability to maintain core body temperature under cold exposure is related to impaired TH activity in the brain^7^,^8^. A modest reduction in body temperature, for instance, prolongs longevity and may delay ageing^32^; in addition, it may combat neurodegenerative disorders^33^ in both poikilothermic and homoeothermic animals. The restoration of cold sensitivity by elevating TH levels in a group of MB afferent neurons suggests that the altered cold avoidance in aged flies is not due to a general failure of the entire dopamine circuit and is not necessarily a drawback.

## Methods

### Fly strains

For *in vivo* calcium imaging and anatomical analysis, fly stocks were raised on cornmeal food^34^ at 25 °C and 70% relative humidity under a 12:12-h light/dark cycle. The following fly lines were used in the current study: *w*^*1118*^ as wild-type flies; *Ddc-GAL4* and *VT19841-GAL4* expressed in PAM-M9 neurons (Supplementary Fig. 9a,b); *0279-GAL4* expressed in PAM-M8 neurons (Supplementary Fig. 9c); *VT57244-GAL4* and *VT30604-GAL4* expressed in MB α′β′-KCs (Supplementary Fig. 9d,e); *VT49246-GAL4* and *VT21845-GAL4* expressed in MB αβ-KCs (Supplementary Fig. 9f,g); *VT41043-GAL4* and *VT44170-GAL4* expressed in MB-M4 neurons (Supplementary Fig. 9h,i); *VT0765-GAL4* and *VT25781-GAL4* expressed in MB-M10 neurons (Supplementary Fig. 9j,k); *L5166-LexA* expressed in the entire MB (Supplementary Fig. 9l); and *UAS-Kir2.1*, *UAS-TNT*, *UAS-ReaChR*, *UAS-TH*, *UAS-TH*^*RNAi*^, *UAS-DopR*^*RNAi*^, *UAS-Dcr2*, *dumb*^*2*^ mutants and *tub-GAL80*^*ts*^ were used to manipulate neural activity. The *tub-GAL80*^*ts*^ line was used to temporally control GAL4 expression to prevent developmental defects caused by the effectors. The *UAS-Kir2.1* line was used to silence neuronal activity^16^, and *UAS-TNT* was used to block neurotransmission^17^. *UAS-ReaChR* was used to optogenetically activate target neurons by a 561-nm laser^30^. The *dumb*^*2*^ mutant was generated by a piggyBac insertion, PBac (WH) DopR^f02676^, with a terminal UAS site for the GAL4-driven misexpression of the adjacent gene^35^. *UAS-GCaMP1.6* (ref. 14) and *LexAop-GCaMP3.0* (ref. 36) were used for *in vivo* calcium imaging. *UAS-mCD8::GFP;UAS-mCD8::GFP* and *LexAop-rCD2::GFP* were used as reporters for GAL4 and LexA expression, and *UAS-Dscam::GFP;UAS-mKO,UAS-mKO;UAS-Syt::HA* was used for polarity analysis. Dscam::GFP acted as a postsynaptic marker^24^, whereas syt::HA served as a presynaptic marker^23^. *LexAop-mKO;+;UAS-CD4::spGFP*_*1–10*_*,LexAop-CD4::spGFP*_*11*_ was used for the GRASP connectivity test^25^. *hs-flp;+;UAS>rCD2,y*^*+*^ *>mCD8::GFP* was used to label single neurons^26^. All VT lines were provided by Barry J. Dickson. For the analysis of dopamine and DopR levels, fly stocks were raised at 25 °C and tested individually for 7–42 days. For behaviour assays, fly stocks were raised at 21, 25 or 30 °C. Wild-type flies for age-dependent behavioural analysis and *dumb*^*2*^-related flies were raised and tested at 25 °C. For Kir2.1 and TNT screening, flies were raised and tested at 25 °C. For adult stage-specific genetic manipulations, flies were raised at 21 °C, transferred to 25 °C for 3 h after eclosion, kept at 30 °C for 5 (*GAL4/tub-GAL80*^*ts*^*;UAS-Kir2.1*) or 7 days (*GAL4;tub-GAL80*^*ts*^*/UAS-TH*^*RNAi*^*;UAS-Dcr2* and *tub-GAL80*^*ts*^*;GAL4/UAS-DopR*^*RNAi*^) and then shifted back to 25 °C for 16 h before the temperature preference assay. For this protocol of temperature shifts, wild-type flies exhibited normal temperature preference (Supplementary Fig. 10). Aged flies were raised at 25 °C, transferred to 30 °C for 5 (*GAL4/tub-GAL80*^*ts*^*;UAS-Kir2.1*) or 7 (*GAL4;tub-GAL80*^*ts*^*/UAS-TH;UAS-TH*) days and then shifted back to 25 °C for 16 h before the temperature preference assay.

### Generation of transgenic flies

pP[LexAop-mKO]: the coding region of *mKO* was amplified by PCR amplification with the forward primer 5′-ATGGTGAGTGTGATTAAA-3′ and the reverse primer 5′-TCAGGAATGATGAGCTACTGC-3′, serving as a template within the plasmid of pmKO1-S1 (MBL, USA). The amplified *mKO* was cloned into pGEM-T-nnn TA cloning vector (Promega, USA). The pGEM-T-easy vector contained a single EcoRI cutting site to release the *mKO* gene, and hence the *mKO* gene was cloned into the EcoRI site of the pP[LOT] vector to create the pP[LexAop-mKO] transgene. The pP[UAS-AI-TH]: pUAST-TH clone was provided by Sean B. Carroll (HHMI). It contained EcoRI and KpnI cutting sites to release the full TH gene, after which this region was cloned into the EcoRI and KpnI sites of the pP[UAST-AI] vector^37^ to create the pP[UAS-AI-TH] transgene.

### Immunohistochemistry

Whole-mount immunolabelling of the adult fly brain was performed as previously described^38^. The brain samples were first dissected in PBS and fixed in 4% paraformaldehyde on ice with two repetitions of microwave irradiation (2,450 MHz, 1,100 Watts) for 90 s with continuous rotation. The samples were then transferred to 4% paraformaldehyde in PBS with 0.25% triton X-100 on ice during two repetitions of 90-s microwave irradiation. Subsequently, the brains were penetrated and blocked in PBS containing 2% Triton X-100 and 10% normal goat serum, and degassed in a vacuum chamber four times to expel tracheal air (depressurized to −70 mm Hg and then held for 10 min). After degassing, the samples were immersed in the same solution for 2 h on a shaker at room temperature (RT). Next, the brains were transferred to the solution with primary antibodies and incubated at RT overnight.

The following primary antibodies were used: mouse 4F3 anti-DLG with 1:100 dilution (developmental studies Hybridoma Bank, University of Iowa), rabbit anti-HA (Abcam) with 1:500 dilution, rabbit anti-GFP (Invitrogen) with 1:500 dilution, mouse anti-TH (ImmunoStar) with 1:200 dilution and rabbit anti-DopR (supplied by F.W. Wolf) with 1:1,250 dilution^39^. After washing in PBS containing 1% Triton X-100 and 3% sodium chloride, the brains were transferred to the solution with secondary antibodies and incubated at RT overnight. The following secondary antibodies were used: biotin-conjugated goat anti-mouse IgGs (Invitrogen) and biotin-conjugated goat anti-rabbit IgGs (Invitrogen), each diluted to 1:200.

Next, the brain samples were washed and incubated in a solution containing 1:500-diluted Alexa Fluor 635 streptavidin (Invitrogen) at RT overnight. Finally, after extensive washing, the immunostained brains were directly cleared and mounted in FocusClear (Celexplorer, Taiwan), an aqueous sugar-based solution that renders biological tissue transparent. Sample brains were imaged under a Zeiss LSM710 confocal microscope with either a × 40 C-Apochromat water-immersion objective lens (numerical aperture value, 1.2; working distance, 220 μm) or a × 63 glycerine immersion objective lens (numerical aperture value, 1.3; working distance, 170 μm).

### Single-neuron analysis

For genetic FLP-out labelling, flies carrying the *VT41043-GAL4/hs-flp;+;UAS>rCD2,y^+^>mCD8::GFP, VT0765-GAL4/hs-flp;+;UAS>rCD2,y^+^>mCD8::GFP, 0279-GAL4/hs-flp;+;UAS>rCD2,y^+^>mCD8::GFP or Ddc-GAL4/hs-flp;+;UAS>rCD2,y^+^>mCD8::GFP* transgenes were heat shocked at the 4-day pupal stage. Individual single-neuron images were recorded with anti-DLG counterstaining. The sample brains were imaged under a Zeiss LSM710 confocal microscope with a × 40 C-Apochromat water immersion objective lens. Individual single-neuron images were recorded, segmented and warped to the standard brain using the software Amira 5.2, as previously described^38^,^40^.

### Behavioural assays

Temperature preference was assayed as previously described^11^,^27^,^41^, with slight modifications. A linear thermal gradient (10–40 °C) was produced by resting an aluminium plate (500 × 300 × 10 mm) on an array of thermoelectric cooling chips (50 × 50 × 2 mm each) at one end and placing either a hot or cold source at the other. The temperatures on the aluminium plate surface were determined using an electro-couple thermometer. Multiple temperature sensors were embedded within the aluminium plate to monitor the temperature gradient. The thermal gradient was divided into 12 sectors. Because the first and last sectors (each 50 mm in width) were the regions where the thermoelectric cooling chips were located underneath, they were constantly kept at 10 and 40 °C, respectively. The region from 10 to 40 °C was divided into 10 sectors (40 mm each), each showing a 3-°C thermal gradient. A 300 × 70-mm copper cuboid with water tunnels was located below the thermoelectric cooling chips. Water flow (20 °C) dissipated the accumulated heat produced by the thermoelectric cooling chips on the side where the copper cuboid was attached. The components mentioned above were fixed on an acrylic base. The glass cover (510 × 310 × 10 mm) included three strips that provided channels to test four groups of flies simultaneously. The glass cover was coated with RainX to prevent flies from escaping the temperature gradient; this did not disturb the normal temperature gradient^27^. For each assay, 80–120 adult flies were subdued with 98% CO_2_ and then loaded at the central regions of the aluminium plate, which was then exposed to the gradient for 30 min in darkness prior to data collection. The experimental results were recorded using a camera, and the number of flies within each thermal sector was calculated offline. All experiments were performed in an environment maintained at 24–26 °C and 40–50% relative humidity. The behavioural data were analysed with Microsoft Excel. Each data point on the curve represents the percentage of flies within that temperature sector (mean±s.e.m.); the temperature value represents the average of that temperature sector. The distributions were compared using a *χ*^2^-test, and the comparisons between experimental groups and each control group were deemed significant if *P*<0.05 (***, all comparisons reached *P*<0.001; **, all comparisons reached *P*<0.01; *, all comparisons reached *P*<0.05; NS, not significant). Values for the avoidance index were compared using a one-tailed *t*-test, and comparisons between experimental groups with wild-type (*w*^*1118*^) as well as either *Kir2.1/w*^*1118*^ or *TNT/w*^*1118*^ of their own effector control groups were deemed significant if *P*<0.05 (***, both comparisons reached *P*<0.001; **, both comparisons reached *P*<0.01; *, both comparisons reached *P*<0.05; NS, not significant).

### Functional imaging

For *in vivo* calcium imaging, flies expressing either GCaMP1.6 or GCaMP3.0 were singly prepared as previously described^28^, with a slight modification. To monitor the calcium changes in response to cold or hot stimuli within the brain of a living fly, we immobilized the fly in a 250-μl pipette tip, opened a window on the head capsule using fine tweezers and immediately added a drop of adult hemolymph-like (AHL) saline (108 mM NaCl, 5 mM KCl, 2 mM CaCl_2_, 8.2 mM MgCl_2_, 4 mM NaHCO_3_, 1 mM NaH_2_PO_4_, 5 mM trehalose, 10 mM sucrose and 5 mM HEPES (pH 7.5, 265 mOsm))^42^ to prevent dehydration. After removing the small trachea and excessive fats with fine tweezers, the pipette tip, along with the fly, was mounted with 200-μl 24-°C AHL solution in a perfusion chamber. The cold stimulus was administered by adding an additional 200 μl of 8–10-°C AHL solution into the chamber. Similarly, the hot stimulus was administered by adding an additional 200 μl of 35–37-°C AHL solution. The final temperature was monitored by a thermometer. Time-lapse recording of changes in GCaMP intensity before and after thermal stimulation was performed on a Zeiss LSM710 microscope with a × 40 Achroplan IR lens. An excitation laser (488 nm) and a GaAsP detector in LSM 710 were used for emissions passing through a 520–550-nm band-pass filter. An optical slice with a resolution of 512 × 512 pixels in the LSM 710 was continuously monitored for 60 s at 2 fps. For the optogenetic experiments, flies were either kept in a standard fly medium or medium containing 400 μM all-*trans*-retinal (Sigma) for 7 days before the calcium imaging assay. Samples were prepared as mentioned earlier; they were fixed in a 250-μl pipette tip with the head capsule opened and a drop of AHL saline placed on the head of the fly. Subsequently, the fly and pipette tip were fixed to a coverslip by tape, and a × 40 water immersion objective (W Plan-Apochromat 40 × /1.0 DIC M27) was used for imaging. A 561-nm laser was used as the light source to activate ReaChR within the region of interest. Five laser pulses were delivered every 20 scans (0.5 s per scan). Two-photon imaging was performed with a titanium:sapphire laser locked at a wavelength of 910 nm, and changes in fluorescence were recorded through a GaAsP detector in Zeiss LSM780. The 495–525-nm GCaMP fluorescence emissions were monitored by a photomultiplier tube. The 910-nm two-photon laser was switched to 561-nm laser while the laser pulse for ReaChR activation was delivered, and then switched back to 910-nm two-photon laser right after the 561-nm laser was shut off. The bleaching/recording setting was applied from the internal program of LSM780. The optogenetic manipulation was spatially specific, because when we activated PAM neurons in one hemisphere, the PAM neurons of the other hemisphere were not responsive (data not shown). The first response of each fly was used for statistical analysis, because the magnitude of each response was similar. A one-tailed *t*-test was used for statistical analysis (***P<0.001; **P<0.01; *P<0.05; NS, not significant). Δ*F*/*F*_0_ intensity maps were generated using ImageJ and Amira 5.2 in all functional calcium imaging studies.

## Additional information

**How to cite this article:** Shih, H.-W. *et al*. Parallel circuits control temperature preference in *Drosophila* during ageing. *Nat. Commun.* 6:7775 doi: 10.1038/ncomms8775 (2015).

## Supplementary Material

Supplementary Information Supplementary Figures 1-10

Supplementary Movie 1 The row data of Figure 7b is displayed.

## References

[b1] Dillon M. E., Wang G., Garrity P. A. & Huey R. B. Review: thermal preference in *Drosophila*. J. Therm. Biol. 34, 109–119 (2009) . 20161211 10.1016/j.jtherbio.2008.11.007 PMC2714919

[b2] Collins K. J., Exton-Smith A. N. & Dore C. Urban hypothermia: preferred temperature and thermal perception in old age. Br. Med. J. 282, 175–177 (1981) . 6779937 10.1136/bmj.282.6259.175 PMC1503968

[b3] Talan M. Age-related changes in thermoregulation of mice. Ann. N. Y. Acad. Sci. 813, 95–100 (1997) . 9100868 10.1111/j.1749-6632.1997.tb51678.x

[b4] Sebastiao A. M., Colino-Oliveira M., Assaife-Lopes N., Dias R. B. & Ribeiro J. A. Lipid rafts, synaptic transmission and plasticity: impact in age-related neurodegenerative diseases. Neuropharmacology 64, 97–107 (2013) . 22820274 10.1016/j.neuropharm.2012.06.053

[b5] Blatteis C. M. Age-dependent changes in temperature regulation - a mini review. Gerontology 58, 289–295 (2012) . 22085834 10.1159/000333148

[b6] Guergova S. & Dufour A. Thermal sensitivity in the elderly: a review. Ageing Res. Rev. 10, 80–92 (2011) . 20685262 10.1016/j.arr.2010.04.009

[b7] Algeri S. . Changes with age in rat central monoaminergic system responses to cold stress. Neurobiol. Aging 3, 237–242 (1982) . 6186926 10.1016/0197-4580(82)90045-8

[b8] Tumer N. & Larochelle J. S. Tyrosine hydroxylase expression in rat adrenal medulla: influence of age and cold. Pharmacol. Biochem. Behav. 51, 775–780 (1995) . 7675858 10.1016/0091-3057(95)00030-z

[b9] Yamamoto A. & Ohba S. Temperature preferences of 11 *Drosophila* species from Japan–the relationship between preferred temperature and some ecological characteristics in their natural habitats. Zoolog. Sci. 4, 631–640 (1984) .

[b10] Neckameyer W. S., Woodrome S., Holt B. & Mayer A. Dopamine and senescence in *Drosophila melanogaster*. Neurobiol. Aging 21, 145–152 (2000) . 10794859 10.1016/s0197-4580(99)00109-8

[b11] Hong S. T. . cAMP signaling in mushroom bodies modulates temperature preference behavior in *Drosophila*. Nature 454, 771–775 (2008) . 18594510 10.1038/nature07090

[b12] Bang S. . Dopamine signaling in mushroom bodies regulates temperature-preference behavior in *Drosophila*. PLoS Genet. 7, e1001346 (2011) . 21455291 10.1371/journal.pgen.1001346 PMC3063753

[b13] White K. E., Humphrey D. M. & Hirth F. The dopaminergic system in the aging brain of *Drosophila*. Front. Neurosci. 4, 205 (2010) . 21165178 10.3389/fnins.2010.00205 PMC3002484

[b14] Nakai J., Ohkura M. & Imoto K. A high signal-to-noise Ca(2+) probe composed of a single green fluorescent protein. Nat. Biotechnol. 19, 137–141 (2001) . 11175727 10.1038/84397

[b15] Yu D., Akalal D. G. & Davis R. L. *Drosophila* α/β mushroom body neurons form a branch-specific, long-term cellular memory trace after spaced olfactory conditioning. Neuron 52, 845–855 (2006) . 17145505 10.1016/j.neuron.2006.10.030 PMC1779901

[b16] Zaritsky J. J., Eckman D. M., Wellman G. C., Nelson M. T. & Schwarz T. L. Targeted disruption of Kir2.1 and Kir2.2 genes reveals the essential role of the inwardly rectifying K(+) current in K(+)-mediated vasodilation. Circ. Res. 87, 160–166 (2000) . 10904001 10.1161/01.res.87.2.160

[b17] Sweeney S. T., Broadie K., Keane J., Niemann H. & O'Kane C. J. Targeted expression of tetanus toxin light chain in *Drosophila* specifically eliminates synaptic transmission and causes behavioral defects. Neuron 14, 341–351 (1995) . 7857643 10.1016/0896-6273(95)90290-2

[b18] Tanaka N. K., Tanimoto H. & Ito K. Neuronal assemblies of the *Drosophila* mushroom body. J. Comp. Neurol. 508, 711–755 (2008) . 18395827 10.1002/cne.21692

[b19] Perisse E., Burke C., Huetteroth W. & Waddell S. Shocking revelations and saccharin sweetness in the study of *Drosophila* olfactory memory. Curr. Biol. 23, R752–R763 (2013) . 24028959 10.1016/j.cub.2013.07.060 PMC3770896

[b20] Perisse E. . Different Kenyon cell populations drive learned approach and avoidance in *Drosophila*. Neuron 79, 945–956 (2013) . 24012007 10.1016/j.neuron.2013.07.045 PMC3765960

[b21] Liu C. . A subset of dopamine neurons signals reward for odour memory in *Drosophila*. Nature 488, 512–516 (2012) . 22810589 10.1038/nature11304

[b22] Burke C. J. . Layered reward signalling through octopamine and dopamine in *Drosophila*. Nature 492, 433–437 (2012) . 23103875 10.1038/nature11614 PMC3528794

[b23] Zhong H., Yokoyama C. T., Scheuer T. & Catterall W. A. Reciprocal regulation of P/Q-type Ca^2+^ channels by SNAP-25, syntaxin and synaptotagmin. Nat. Neurosci. 2, 939–941 (1999) . 10526329 10.1038/14721

[b24] Schmucker D. . *Drosophila* Dscam is an axon guidance receptor exhibiting extraordinary molecular diversity. Cell 101, 671–684 (2000) . 10892653 10.1016/s0092-8674(00)80878-8

[b25] Feinberg E. H. . GFP Reconstitution Across Synaptic Partners (GRASP) defines cell contacts and synapses in living nervous systems. Neuron 57, 353–363 (2008) . 18255029 10.1016/j.neuron.2007.11.030

[b26] Wong A. M., Wang J. W. & Axel R. Spatial representation of the glomerular map in the *Drosophila* protocerebrum. Cell 109, 229–241 (2002) . 12007409 10.1016/s0092-8674(02)00707-9

[b27] Hamada F. N. . An internal thermal sensor controlling temperature preference in *Drosophila*. Nature 454, 217–220 (2008) . 18548007 10.1038/nature07001 PMC2730888

[b28] Gallio M., Ofstad T. A., Macpherson L. J., Wang J. W. & Zuker C. S. The coding of temperature in the *Drosophila* brain. Cell 144, 614–624 (2011) . 21335241 10.1016/j.cell.2011.01.028 PMC3336488

[b29] McGuire S. E., Le P. T., Osborn A. J., Matsumoto K. & Davis R. L. Spatiotemporal rescue of memory dysfunction in *Drosophila*. Science 302, 1765–1768 (2003) . 14657498 10.1126/science.1089035

[b30] Inagaki H. K. . Optogenetic control of *Drosophila* using a red-shifted channelrhodopsin reveals experience-dependent influences on courtship. Nat. Methods 11, 325–332 (2014) . 24363022 10.1038/nmeth.2765 PMC4151318

[b31] Backman L., Nyberg L., Lindenberger U., Li S. C. & Farde L. The correlative triad among aging, dopamine, and cognition: current status and future prospects. Neurosci. Biobehav. Rev. 30, 791–807 (2006) . 16901542 10.1016/j.neubiorev.2006.06.005

[b32] Conti B. Considerations on temperature, longevity and aging. Cell. Mol. Life Sci. 65, 1626–1630 (2008) . 18425417 10.1007/s00018-008-7536-1 PMC2574693

[b33] Salerian A. J. & Saleri N. G. Cooler biologically compatible core body temperatures may prolong longevity and combat neurodegenerative disorders. Med. Hypotheses 66, 636–642 (2006) . 16326025 10.1016/j.mehy.2005.07.021

[b34] Cline T. W. Two closely linked mutations in *Drosophila melanogaster* that are lethal to opposite sexes and interact with daughterless. Genetics 90, 683–698 (1978) . 105964 10.1093/genetics/90.4.683 PMC1213913

[b35] Thibault S. T. . A complementary transposon tool kit for *Drosophila melanogaster* using P and piggyBac. Nat. Genet. 36, 283–287 (2004) . 14981521 10.1038/ng1314

[b36] Pfeiffer B. D., Truman J. W. & Rubin G. M. Using translational enhancers to increase transgene expression in *Drosophila*. Proc. Natl Acad. Sci. USA 109, 6626–6631 (2012) . 22493255 10.1073/pnas.1204520109 PMC3340069

[b37] Kuo S. Y. . A hormone receptor-based transactivator bridges different binary systems to precisely control spatial-temporal gene expression in *Drosophila*. PLoS ONE 7, e50855 (2012) . 23239992 10.1371/journal.pone.0050855 PMC3519826

[b38] Shih H. W. & Chiang A. S. Anatomical characterization of thermosensory AC neurons in the adult *Drosophila* brain. J. Neurogenet. 25, 1–6 (2011) . 21510718 10.3109/01677063.2011.571323

[b39] Kong E. C. . A pair of dopamine neurons target the D1-like dopamine receptor DopR in the central complex to promote ethanol-stimulated locomotion in *Drosophila*. PLoS ONE 5, e9954 (2010) . 20376353 10.1371/journal.pone.0009954 PMC2848596

[b40] Chiang A. S. . Three-dimensional reconstruction of brain-wide wiring networks in *Drosophila* at single-cell resolution. Curr. Biol. 21, 1–11 (2011) . 21129968 10.1016/j.cub.2010.11.056

[b41] Sayeed O. & Benzer S. Behavioral genetics of thermosensation and hygrosensation in *Drosophila*. Proc. Natl Acad. Sci. USA 93, 6079–6084 (1996) . 8650222 10.1073/pnas.93.12.6079 PMC39192

[b42] Wang J. W., Wong A. M., Flores J., Vosshall L. B. & Axel R. Two-photon calcium imaging reveals an odor-evoked map of activity in the fly brain. Cell 112, 271–282 (2003) . 12553914 10.1016/s0092-8674(03)00004-7

