# Supplementary material for: Parallel circuits control temperature preference in Drosophila during ageing
Source: Nat Commun. 2015 Jul 16;6:7775. doi: 10.1038/ncomms8775 (PMC4518306; doi:10.1038/ncomms8775)
Supplement: Supplementary Information — Supplementary Figures 1-10 [file ncomms8775-s1.pdf]

Supplementary Material

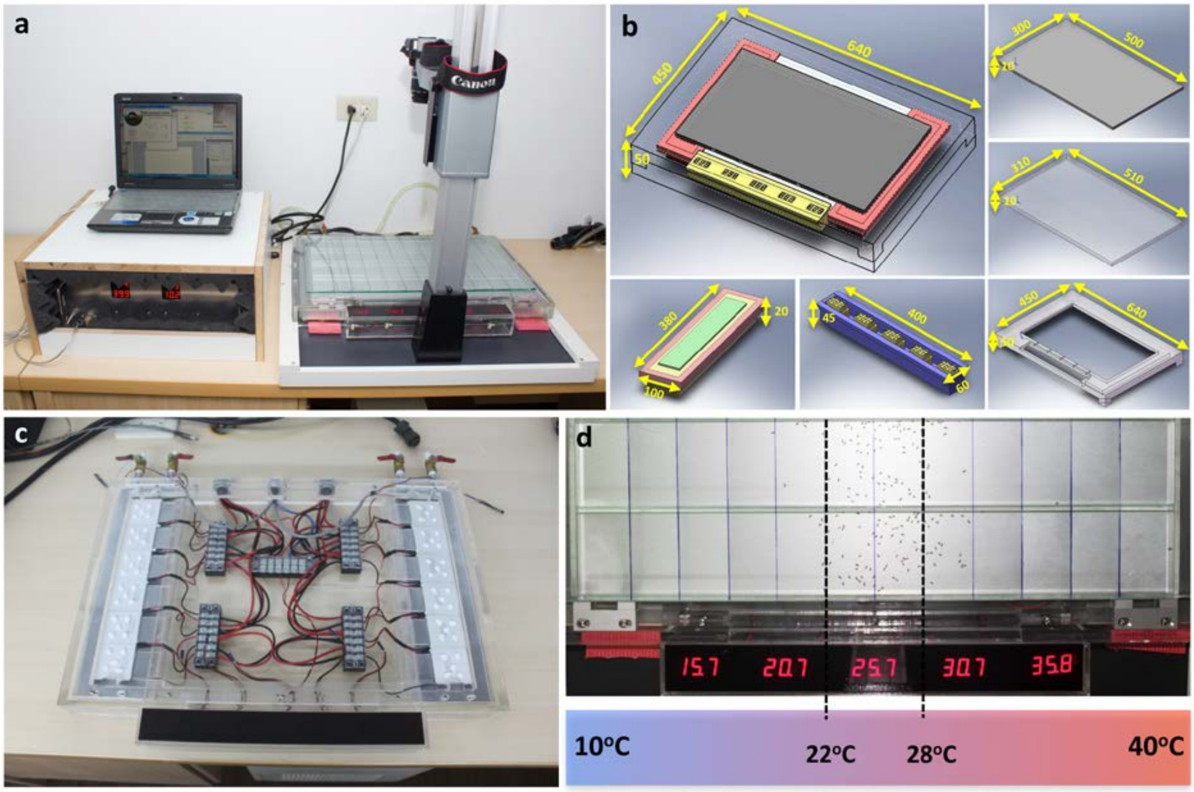

Supplementary Figure 1 Behavioral assay for temperature preference. (**a**) Photograph of system composed of thermal gradient plate, camera, computer, and control box. (**b**) Thermal gradient plate includes temperature control device composed of an array of thermoelectric cooling chips (green) with copper block (yellow), monitoring sensors, aluminum plate, glass cover, and acrylic base. Unit, mm. (**c**) Arrays of cooling chips are wired together to receive commands from controller and adjust temperature. (**d**) Population distribution of wild-type flies (w^{1118}) on thermal gradient plate with temperature sensors.

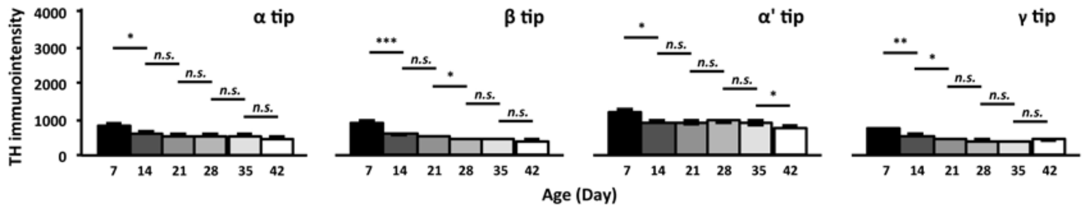

Supplementary Figure 2 Age-dependent changes of brain tyrosine hydroxylase (TH) levels in different MB lobes. Fluorescent intensity of TH-immunopositive signals in each MB lobe was measured for flies at different ages. Values represent mean ± s.e.m. (n = 12). ***, $P < 0.001$; **, $P < 0.01$; *, $P < 0.05$; *n.s.*, not significant.

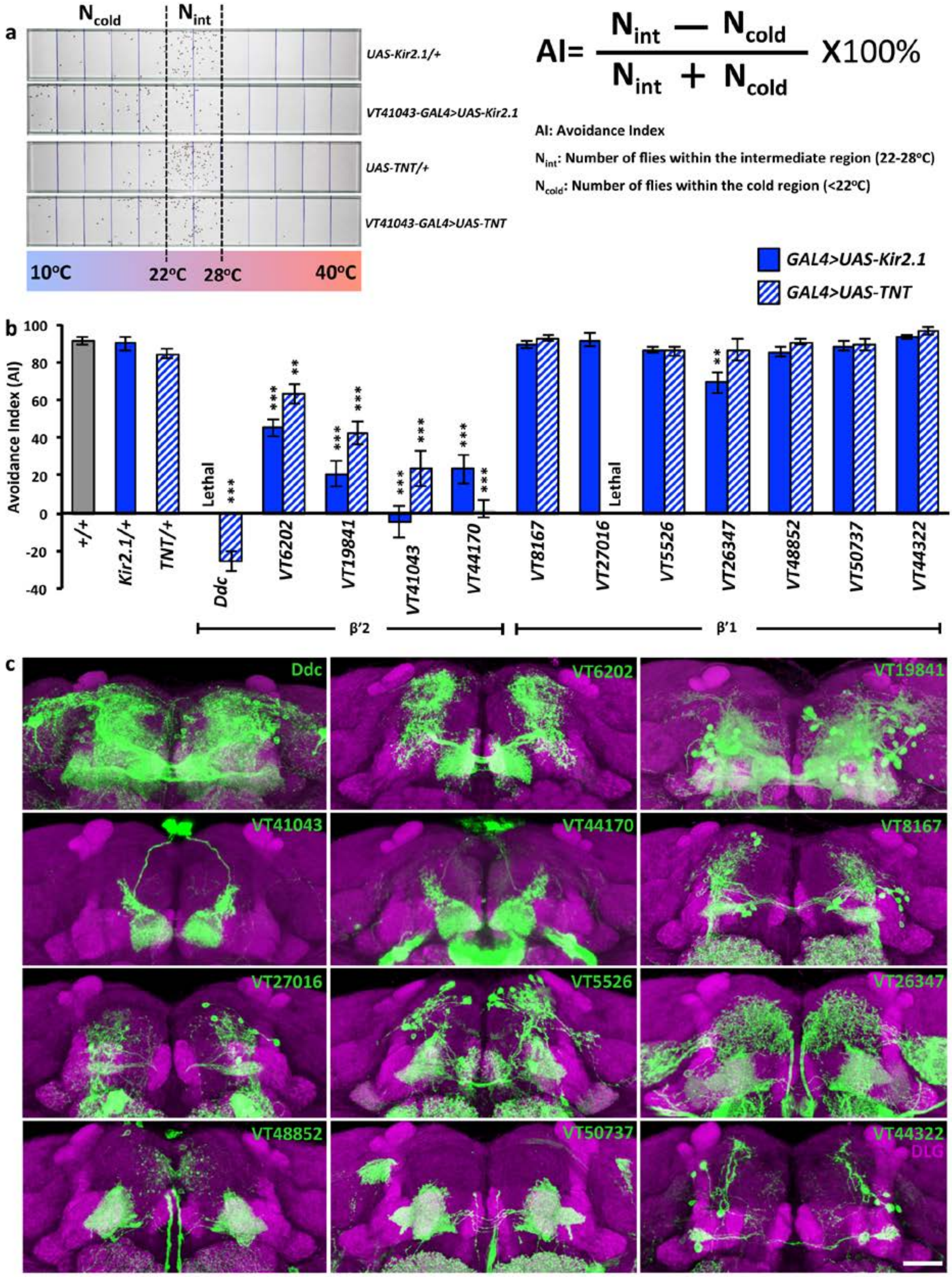

Supplementary Figure 3 Behavioral screening of GAL4 lines containing candidate MB β′ extrinsic neurons involved in cold avoidance. (**a**) Photograph of control and manipulated flies distributed along temperature gradient. Right, formula for calculating cold avoidance

index (AI)[11]. (**b**) Effects of silencing GAL4 neurons by Kir2.1 or TNT on cold avoidance. β′ innervation can be divided into proximal β′1 and distal β′2 segments[18]. (**c**) GFP expression in the MB driven by various GAL4 lines. Brain neuropils were counterstained with anti-DLG immunostaining (magenta). GFP signals show sectional projections to illustrate innervation patterns in the β′ lobe, whereas DLG signals show whole-brain projections. Scale bar, 20 μm. Values represent mean ± s.e.m. ($n \geq 8$). ***, $P < 0.001$; **, $P < 0.01$. Genotypes: (1) +/+, (2) *UAS-Kir2.1/+*, (3) *UAS-TNT/+*, (4) *GAL4/UAS-Kir2.1*, (5) *GAL4/UAS-TNT*, and (6) *GAL4/UAS-mCD8::GFP;UAS-mCD8::GFP.*

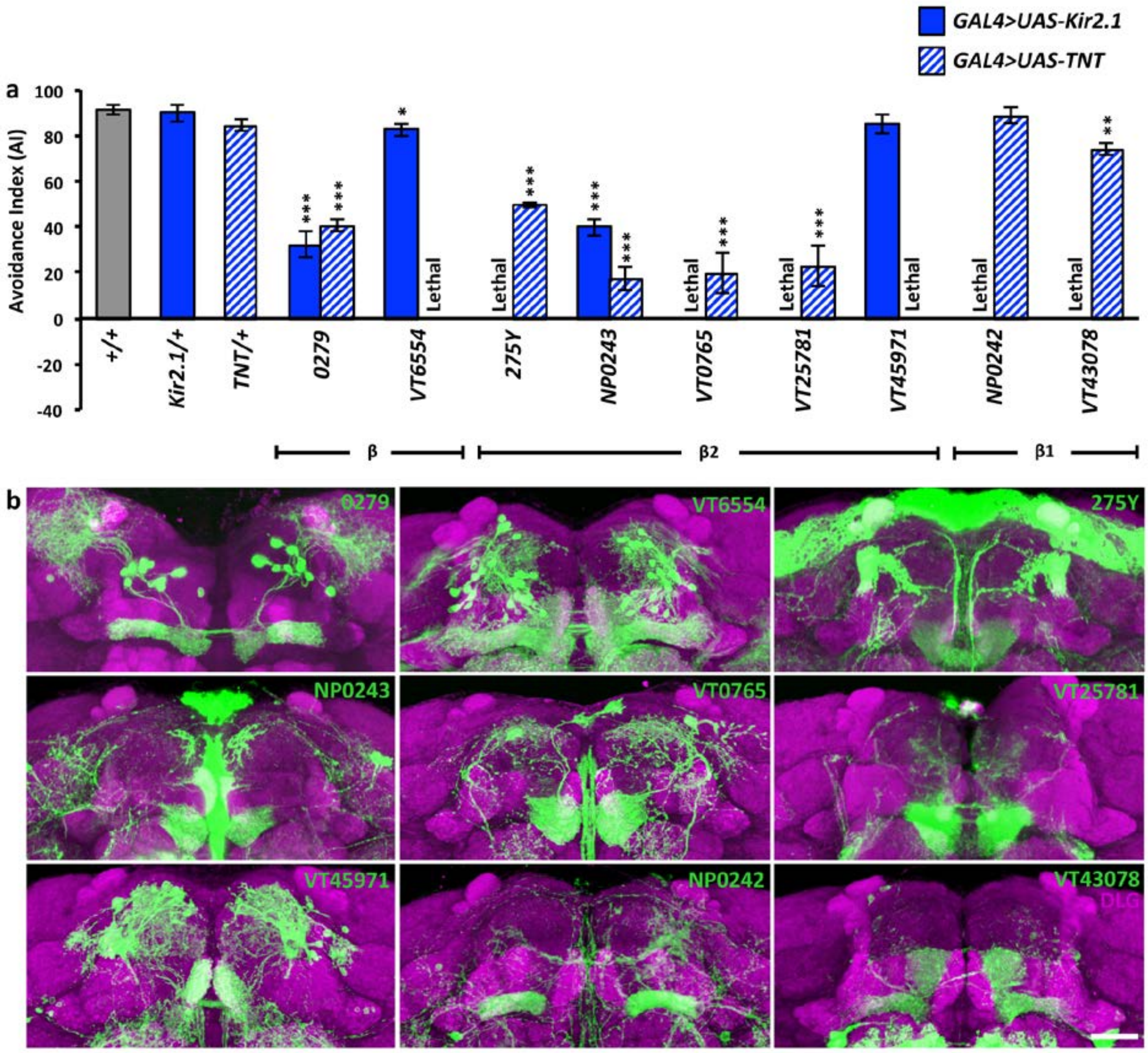

Supplementary Figure 4 Behavioral screening of GAL4 lines containing candidate MB β extrinsic neurons involved in cold avoidance. (**a**) Effects of silencing GAL4 neurons by Kir2.1 or TNT on cold avoidance. β innervation can be catalogued into entire β lobe, proximal β1 and distal β2 segments[18]. (**b**) GFP expression in the MB driven by various GAL4 lines. Brain neuropils were counterstained with anti-DLG immunostaining (magenta). GFP signals show sectional projections to illustrate innervation patterns in β lobe, whereas DLG signals show whole-brain projections. Scale bar, 20 μm. Values represent mean ± s.e.m. ($n \geq$ 8). ***, *P < 0.001*; **, *P < 0.01*; *, *P < 0.05*. Genotypes: (1) +/+, (2) *UAS-Kir2.1*/+, (3) *UAS-TNT*/+, (4) *GAL4/UAS-Kir2.1*, (5) *GAL4/UAS-TNT*, and (6) *GAL4/UAS-mCD8::GFP;UAS-mCD8::GFP.*

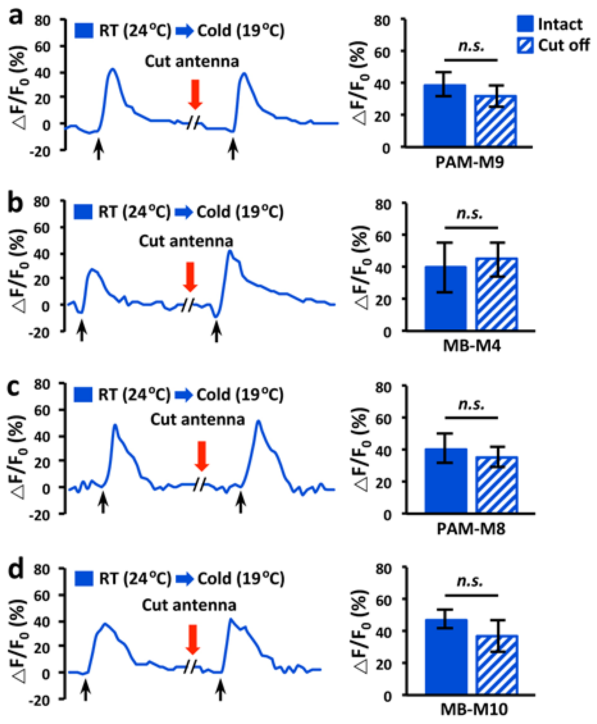

Supplementary Figure 5 Functional imaging of β′ and β circuits in response to cold stimuli with or without intact antennae. Temporal changes in GCaMP1.6 response of PAM-M9 neurons (n = 10) (**a**), MB-M4 neurons (n = 4) (**b**), PAM-M8 neurons (n = 8) (**c**), and MB-M10 neurons (n = 4) (**d**) before and after cutting off the antennae. The arrows indicate the time points at which the temperature stimulus was applied. Right, quantification of changes in GCaMP1.6 fluorescence ($\Delta F/F_0$) in response to cold stimuli. *n.s.*, not significant. Genotypes: (1) *Ddc-GAL4/UAS-GCaMP1.6*, (2) *VT41043-GAL4/UAS-GCaMP1.6*, (3) *0279-GAL4/UAS-GCaMP1.6*, and (4) *VT0765-GAL4/UAS-GCaMP1.6*.

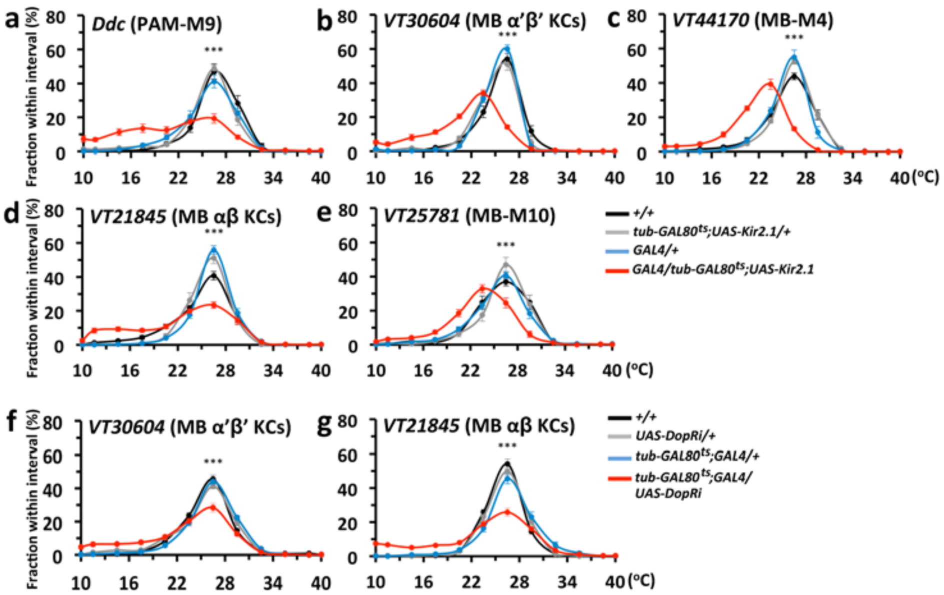

Supplementary Figure 6 Neural activities in both β′ and β circuits contribute to normal temperature preference in young flies. (**a**–**e**) Inhibiting neural activity in PAM-M9 neurons under *Ddc-GAL4* expression (**a**), MB α′β′ KCs under *VT30604-GAL4* expression (**b**), MB-M4 under *VT44170-GAL4* expression (**c**), MB αβ KCs under *VT21845-GAL4* expression (**d**), and MB-M10 under *VT25781-GAL4* expression (**e**) impaired cold avoidance. (**f**,**g**) Downregulating DopR levels in α′β′ KCs under *VT30604-GAL4* (**f**) or in αβ KCs under *VT21845-GAL4* (**g**) impaired cold avoidance. Values represent mean ± s.e.m. ($n \geq 12$). ***, $P < 0.001$. Genotypes: (1) +/+, (2) *tub-GAL80*^{ts}*;UAS-Kir2.1/+*, (3) *GAL4/+*, (4) *Ddc-GAL4/tub-GAL80*^{ts}*;UAS-Kir2.1*, (5) *VT30604-GAL4/tub-GAL80*^{ts}*;UAS-Kir2.1*, (6) *VT44170-GAL4/tub-GAL80*^{ts}*;UAS-Kir2.1*, (7) *VT21845-GAL4/tub-GAL80*^{ts}*;UAS-Kir2.1*, (8) *VT25781-GAL4/tub-GAL80*^{ts}*;UAS-Kir2.1*, (9) *UAS-DopR*^{RNAi}/+, (10) *tub-GAL80*^{ts}*;VT30604-GAL4*/+, (11) *tub-GAL80*^{ts}*;VT30604-GAL4*/*UAS-DopR*^{RNAi}, (12) *tub-GAL80*^{ts}*;VT21845-GAL4*/+, and (13) *tub-GAL80*^{ts}*;VT21845-GAL4*/*UAS-DopR*^{RNAi}.

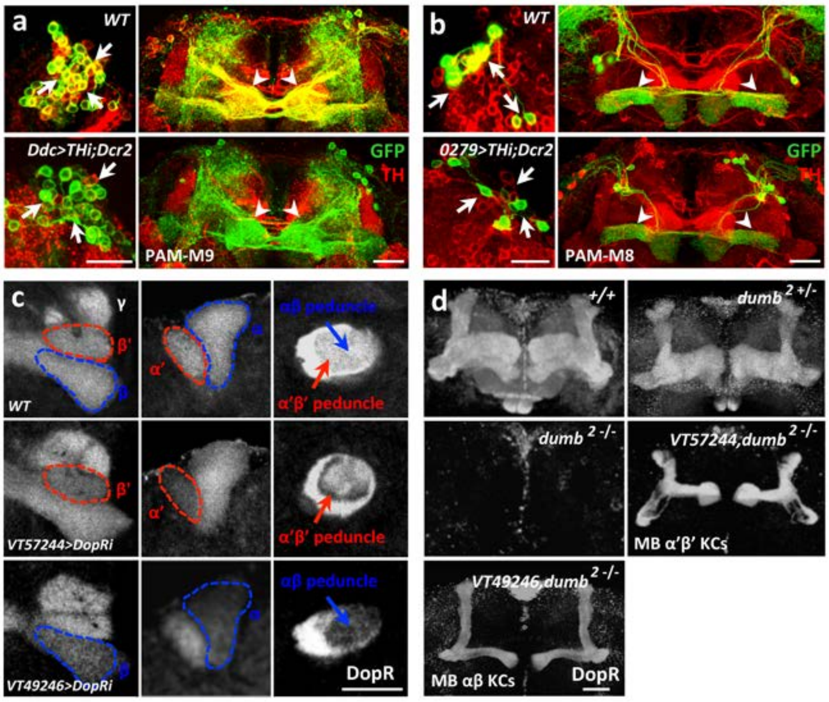

Supplementary Figure 7 Immunohistochemical validation of *UAS-TH*^{RNAi}, *UAS-DopR*^{RNAi}, and *dumb*^{2} mutants. (**a**,**b**) Anti-TH immunopositive cell bodies (arrows) and terminals (arrowheads) of PAM-M9 neurons (**a**, upper panel) and PAM-M8 neurons (**b**, upper panel). Effectiveness and specificity of *Ddc-GAL4>UAS-TH*^{RNAi}*;UAS-Dcr2* and *0279-GAL4>UAS-TH*^{RNAi}*;UAS-Dcr2* were validated by reduced anti-TH immunopositive signals (red) in PAM-M9 (**a**, lower panels) and PAM-M8 neurons (**b**, lower panels). (**c**) Anti-DopR immunopositive signals in MBs (upper panels). Effectiveness and specificity of *VT57244-GAL4>UAS-DopR*^{RNAi} or *VT49246-GAL4>UAS-DopR*^{RNAi} were validated by reduced anti-DopR immunopositive signals (gray) in α′β′ or αβ KCs at α′β′ lobe, αβ lobe, or peduncle (α′β′ KCs: red arrows; αβ KCs: blue arrows). (**d**) Anti-DopR immunopositive signals (gray) were strong in all MB lobes in wild-type flies, mildly reduced in heterozygote *dumb*^{2} mutants (*dumb*$^{2+/-}$), and undetectable in homozygote *dumb*^{2} mutants (*dumb*$^{2-/-}$). *VT57244-GAL4>dumb*^{2} homozygote mutants (*VT57244,dumb*$^{2-/-}$) or *VT49246-GAL4>dumb*^{2} homozygote mutants (*VT49246,dumb*$^{2-/-}$) showed restored anti-DopR immunopositive signals exclusively in α′β′ KCs or αβ KCs. Scale bar, 20 μm. Genotypes: (1) *Ddc-GAL4/UAS-*

mCD8::GFP, (2) *Ddc-GAL4;UAS-mCD8::GFP/UAS-TH*^{RNAi}*;UAS-Dcr2*, (3) *0279-GAL4/UAS-mCD8::GFP*, (4) *UAS-mCD8::GFP;0279-GAL4/UAS-TH*^{RNAi}*;UAS-Dcr2,* (5) +/+, (6) *VT57244-GAL4/UAS-DopR*^{RNAi}, (7) *VT49246-GAL4/UAS-DopR*^{RNAi}, (8) *dumb*^{2}/+, (9) *dumb*^{2}*/dumb*^{2}, (10) *VT57244-GAL4,dumb*^{2}*/VT57244-GAL4,dumb*^{2}, and (11) *VT49246-GAL4,dumb*^{2}*/VT49246-GAL4,dumb*^{2}.

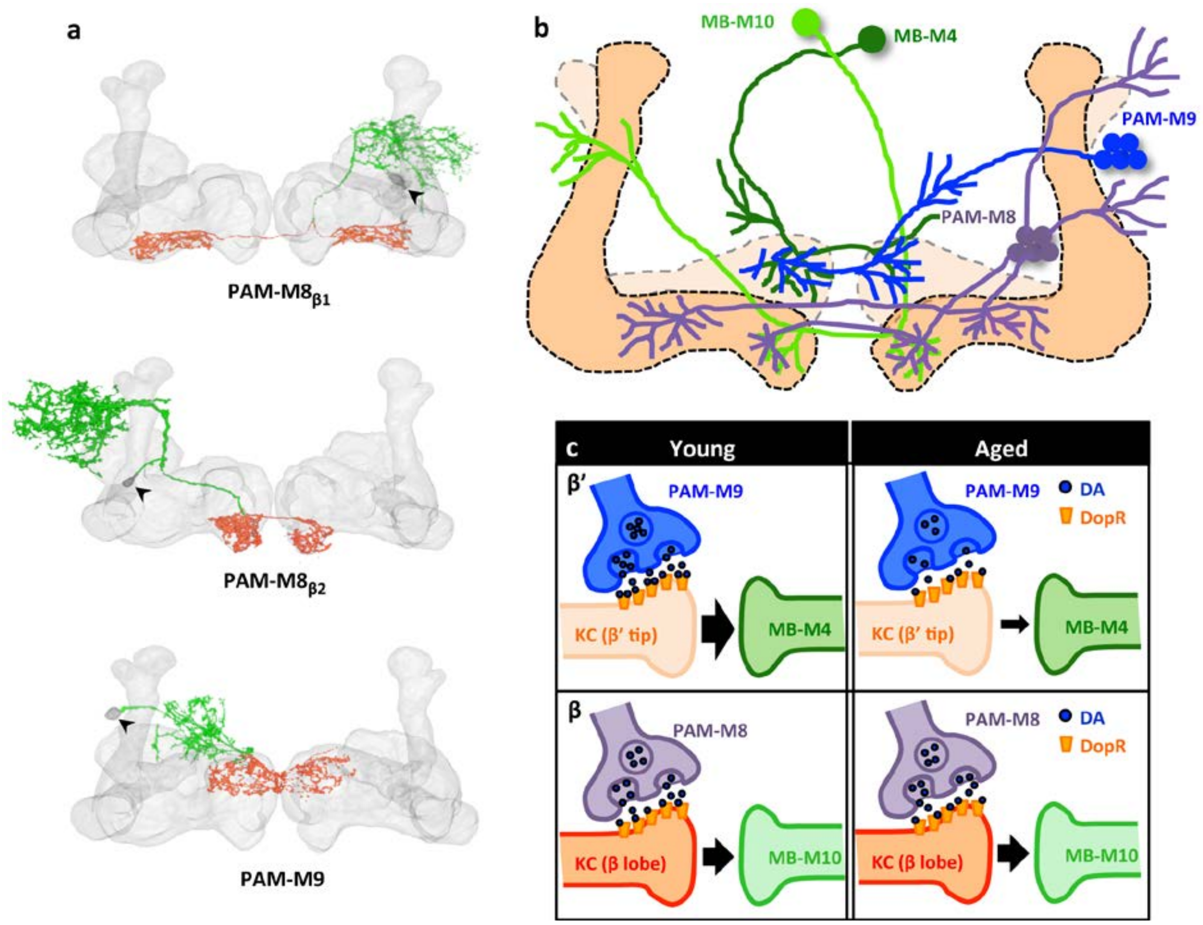

Supplementary Figure 8 A model showing the role of the two parallel brain circuits in regulating temperature preference during the course of aging in fruit flies. (**a**) Morphology and innervation patterns of individual PAM-M8 (composed of PAM-M8$_{\beta1}$ and PAM-M8$_{\beta2}$) and PAM-M9 neurons in the context of the MB volume model. Individual PAM-M8 and PAM-M9 neurons were derived from flip-out labeling of *0279-GAL4* and *Ddc-GAL4*, respectively. Dendrites (green) and axons (red) were manually assigned. Arrowheads indicate cell bodies. (**b**) Schematic illustration of β′ and β circuits containing the PAM-M9→α′β′ KCs→MB-M4 and PAM-M8→ αβ KCs→MB-M10 pathway, respectively (see **Fig. 3** for polarity, connectivity, and morphology of MB-M4 and MB-M10 neurons). (**c**) Age-dependent changes in the contribution of the β′ and β circuits. In young flies, the β′ circuit plays a more important role than the β circuit in maintaining temperature preference through dopamine signaling. However, as flies age, the dopamine level in PAM-M9 neurons

dramatically decreases, weakening the functioning of the β′ circuit, while the functioning of the β circuit remains stable. Therefore, the β circuit takes over the role of maintaining cold avoidance in aged flies. Genotypes: (1) *0279-GAL4/hs-flp;+;UAS>rCD2,y*$^{+}$*>mCD8::GFP*, and (2) *Ddc-GAL4/hs-flp;+;UAS>rCD2,y*$^{+}$*>mCD8::GFP.*

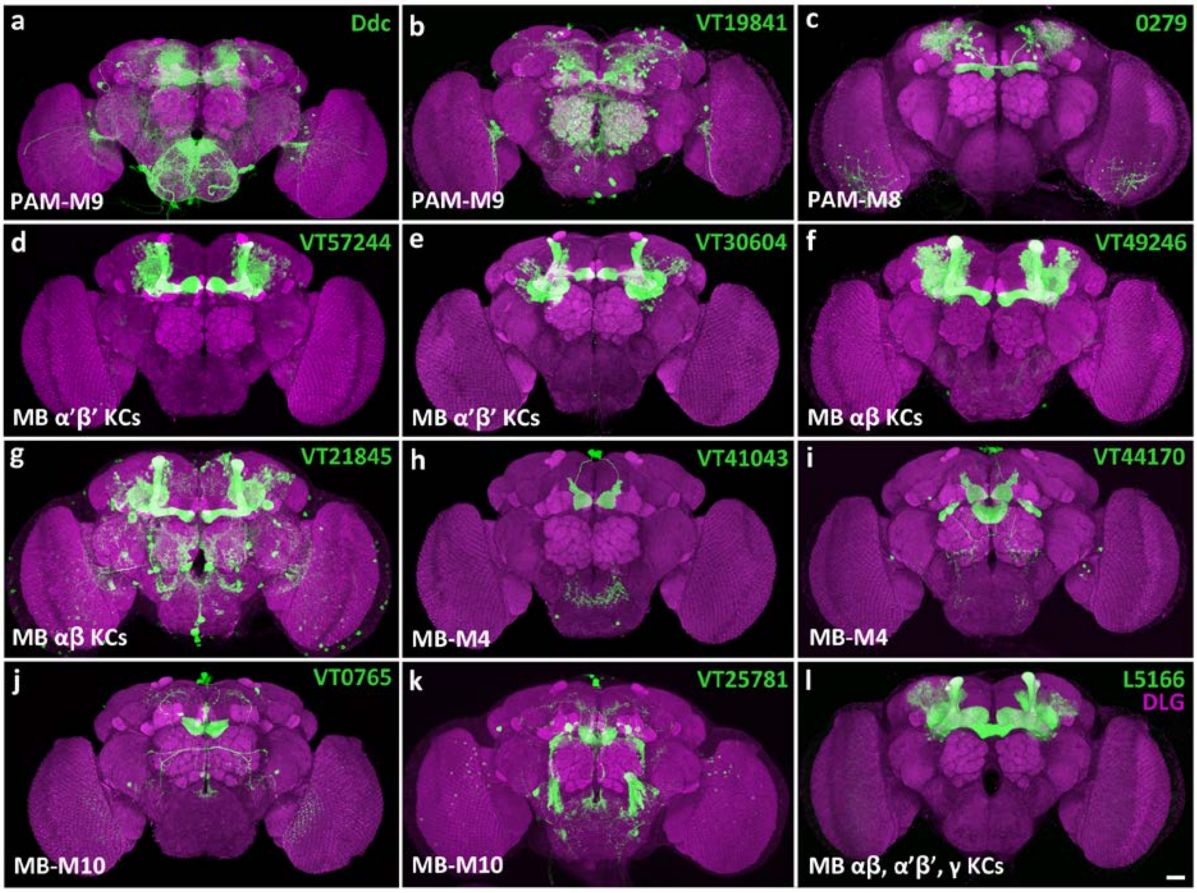

Supplementary Figure 9 GFP expression patterns driven by GAL4 and LexA lines used in this study. (**a**,**b**) PAM-M9 neurons under *Ddc-GAL4* (**a**) and *VT19841-GAL4* (**b**) expression. (**c**) PAM-M8 neurons under *0279-GAL4* expression. (**d**,**e**) α′β′ KCs under *VT57244-GAL4* (**d**) and *VT30604-GAL4* (**e**) expression. (**f**,**g**) αβ KCs under *VT49246-GAL4* (**f**) and *VT21845-GAL4* (**g**) expression. (**h**,**i)** MB-M4 neurons under *VT41043-GAL4* (**h**) and *VT44170-GAL4* (**i**) expression. (**j**,**k**) MB-M10 neurons under *VT0765-GAL4* (**j**) and *VT25781-GAL4* (**k**) expression. (**l**), All MB KCs under *L5166-LexA* expression. Brain neuropils were counterstained with anti-DLG immunostaining (magenta). Scale bar, 20 μm. Genotypes: (1) *Ddc-GAL4/UAS-mCD8::GFP;UAS-mCD8::GFP*, (2) *VT19841-GAL4/UAS-mCD8::GFP;UAS-mCD8::GFP*, (3) *0279-GAL4/UAS-mCD8::GFP;UAS-mCD8::GFP*, (4) *VT57244-GAL4/UAS-mCD8::GFP;UAS-mCD8::GFP*, (5) *VT30604-GAL4/UAS-mCD8::GFP;UAS-mCD8::GFP*, (6) *VT49246-GAL4/UAS-mCD8::GFP;UAS-mCD8::GFP*,

(7) *VT21845-GAL4/UAS-mCD8::GFP;UAS-mCD8::GFP*, (8) *VT41043-GAL4/UAS-mCD8::GFP;UAS-mCD8::GFP*, (9) *VT44170-GAL4/UAS-mCD8::GFP;UAS-mCD8::GFP*, (10) *VT0765-GAL4/UAS-mCD8::GFP;UAS-mCD8::GFP*, (11) *VT25781-GAL4/ UAS-mCD8::GFP;UAS-mCD8::GFP*, and (12) *L5166-LexA/LexAop-rCD2::GFP*.

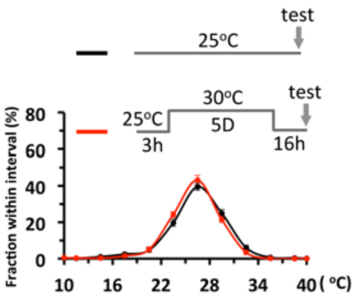

Supplementary Figure 10 Normal temperature preference after chronic temperature shifts. Wild-type flies were raised under a constant temperature of either 25 °C (black) or 21 °C during embryonic and larval development, transferred to 25 °C for 3 h after adult eclosion, maintained at 30 °C for 5 days, and then shifted back to 25 °C for 16 h before the temperature preference assay was conducted (red). Values represent mean ± s.e.m. ($n \geq 8$).

Supplementary Movie

In this movie, the row data of Figure 7b is displayed. The experiment was designed to test the optogenetic condition in our set up is capable to activate PAM neurons. We expressed both GCaMP1.6 and ReaChR under *Ddc-GAL4*. When we activated a portion of the *Ddc-GAL4* neurons (dotted blue line) with 561-nm laser five times with 20-scan intervals (0.5s per scan), the GCaMP1.6 fluorescence intensity increased dramatically in the β′ tip of *Ddc-GAL4* neurons (dotted red line) right after the 561-nm laser delivery. This temporally, spatially specific manipulation through optogenetics is capable to activate targeted neurons.

Genotypes

Fig. 1. Genotype: +/+.

Fig. 2. Genotypes: *OK107-GAL4/UAS-GCaMP1.6.*

Fig. 3. Genotypes: (1) *VT41043-GAL4/UAS-Dscam::GFP;UAS-mKO,UAS-mKO;UAS-syt::HA*, (2) *VT0765-GAL4/UAS-Dscam::GFP;UAS-mKO,UAS-mKO;UAS-syt::HA*, (3) *L0124-LexA;VT41043-GAL4/LexAop-mKO;+;UAS-CD4::spGFP*$_{1\text{-}10}$*,LexAop-CD4::spGFP*$_{11}$, (4) *L0124-LexA;VT0765-GAL4/LexAop-mKO;+;UAS-CD4::spGFP*$_{1\text{-}10}$*,LexAop-CD4::spGFP*$_{11}$, (5) *VT41043-GAL4/hs-flp;+;UAS>rCD2,y*$^{+}$*>mCD8::GFP*, and (6) *VT0765-GAL4/hs-flp;+;UAS>rCD2,y*$^{+}$*>mCD8::GFP*.

Fig. 4. Genotypes: (1) *Ddc-GAL4/UAS-GCaMP1.6*, (2) *VT57244-GAL4/UAS-GCaMP1.6*, (3) *VT41043-GAL4/UAS-GCaMP1.6*, (4) *0279-GAL4/UAS-GCaMP1.6*, (5) *VT49246-GAL4/ UAS-GCaMP1.6*, and (6) *VT0765-GAL4/UAS-GCaMP1.6.*

Fig. 5. Genotypes: (1) +/+, (2) *tub-GAL80*^{ts}*;UAS-Kir2.1/+*, (3) *GAL4/+*, (4) *VT19841-GAL4/tub-GAL80*^{ts}*;UAS-Kir2.1*, (5) *VT57244-GAL4/tub-GAL80*^{ts}*;UAS-Kir2.1*, (6) *VT41043-GAL4/tub-GAL80*^{ts}*;UAS-Kir2.1*, (7) *0279-GAL4/tub-GAL80*^{ts}*;UAS-Kir2.1*, (8) *VT49246-GAL4/tub-GAL80*^{ts}*;UAS-Kir2.1*, (9) *VT0765-GAL4/tub-GAL80*^{ts}*;UAS-Kir2.1*, (10) *UAS-TH*^{RNAi}*;UAS-Dcr2/+*, (11) *Ddc-GAL4;tub-GAL80*^{ts}*/+*, (12) *Ddc-GAL4;tub-GAL80*^{ts}*/UAS-TH*^{RNAi}*;UAS-Dcr2*, (13) *tub-GAL80*^{ts}*;VT19841-GAL4/+*, (14) *tub-GAL80*^{ts}*;VT19841-*

GAL4/UAS-TH^{RNAi};UAS-Dcr2, (15) *tub-GAL80^{ts};0279-GAL4/+*, (16) *tub-GAL80^{ts};0279-GAL4/UAS-TH^{RNAi};UAS-Dcr2*, (17) *UAS-DopR^{RNAi}/+*, (18) *tub-GAL80^{ts};VT57244-GAL4/+*, (19) *tub-GAL80^{ts};VT57244-GAL4/UAS-DopR^{RNAi}*, (20) *tub-GAL80^{ts};VT49246-GAL4/+*, (21) *tub-GAL80^{ts};VT49246-GAL4/UAS-DopR^{RNAi}*, (22) *dumb^{2}/+*, (23) *dumb^{2}/dumb^{2}*, (24) *VT57244-GAL4,dumb^{2}/VT57244-GAL4,dumb^{2}*, and (25) *VT49246-GAL4,dumb^{2}/VT49246-GAL4,dumb^{2}*.

Fig. 6. Genotypes: (1) *L5166-LexA;LexAop-GCaMP3.0/+*, (2) *Ddc-GAL4;UAS-TH;UAS-TH/ L5166-LexA;LexAop-GCaMP3.0*, (3) +/+, (4) *Ddc-GAL4;tub-GAL80^{ts}/UAS-TH;UAS-TH*, (5) *tub-GAL80^{ts};VT19841-GAL4/UAS-TH;UAS-TH*, (6) *VT57244-GAL4/tub-GAL80^{ts};UAS-Kir2.1*, and (7) *VT49246-GAL4/tub-GAL80^{ts};UAS-Kir2.1*.

Fig. 7. Genotypes: (1) *L5166-LexA;LexAop-GCaMP3.0/+*, (2) *Ddc-GAL4;tub-GAL80^{ts}/L5166-LexA;LexAop-GCaMP3.0*, (3) *tub-GAL80^{ts};UAS-Kir2.1/L5166-LexA;LexAop-GCaMP3.0*, (4) *Ddc-GAL4;tub-GAL80^{ts};UAS-Kir2.1/L5166-LexA;LexAop-GCaMP3.0*, (5) *Ddc-GAL4;UAS-GCaMP1.6/UAS-ReaChR*, (6) *Ddc-GAL4/UAS-GCaMP1.6*, (7) *Ddc-GAL4/L5166-LexA;LexAop-GCaMP3.0*, (8) *UAS-ReaChR/L5166-LexA;LexAop-GCaMP3.0* and (9) *Ddc-GAL4;+;UAS-ReaChR/L5166-LexA;LexAop-GCaMP3.0.*
